# Antimicrobial Activity of Selected Antimicrobial Peptides Against Planktonic Culture and Biofilm of *Acinetobacter baumannii*

**DOI:** 10.1007/s12602-018-9444-5

**Published:** 2018-07-24

**Authors:** Maciej Jaśkiewicz, Damian Neubauer, Kamil Kazor, Sylwia Bartoszewska, Wojciech Kamysz

**Affiliations:** Department of Inorganic Chemistry, Faculty of Pharmacy, Medical University of Gdansk, Gdansk, Poland

**Keywords:** *Acinetobacter baumannii*, Antibiotics, Antimicrobial peptides, Antimicrobial susceptibility testing, Biofilm, Peptide drugs, Tracheal tubes

## Abstract

*Acinetobacter baumannii* is one of the most challenging pathogens, on account of its predisposition to develop resistance leading to severe, difficult-to-treat infections. As these bacteria are more usually isolated from nosocomial infections, the new therapeutic options are demanded. Antimicrobial peptides (AMPs) are compounds likely to find application in the treatment of *A. baumannii*. These compounds exhibit a wide spectrum of antimicrobial activity and were found to be effective against biofilm. In this study, eight AMPs, namely aurein 1.2, CAMEL, citropin 1.1., LL-37, omiganan, r-omiganan, pexiganan, and temporin A, were tested for their antimicrobial activity. A reference strain of *A. baumannii* ATCC 19606 was used. Antimicrobial assays included determination of the minimum inhibitory concentration and the minimum biofilm eradication concentration. Considering the fact that the majority of *A. baumannii* infections are associated with mechanical ventilation and the use of indwelling devices, the activity against biofilm was assessed on both a polystyrene surface and tracheal tube fragments. In addition, cytotoxicity (HaCaT) was determined and in vitro selectivity index was calculated.

## Introduction

The *Acinetobacter* bacteria are currently one of the major causes of nosocomial infections while *Acinetobacter baumannii* is considered to be the major pathogen, owing to its predisposition to develop resistance [1]. These aerobic, Gram-negative bacteria have been routinely isolated form nosocomial infections, notably from patients of intensive care units [2]. These infections are frequently severe and difficult to treat, on account of their correlation with inter-regional spread and local occurrence of *A. baumannii* resistant to carbapenems [3]. This fact is still more alarming since these β-lactam antibiotics are considered to be the last-line remedy used in the treatment of patients infected with multi-resistant Gram-negative bacteria [4]. The *A. baumannii* causes a wide variety of infections, but most of them are associated with the respiratory tract and the use of indwelling devices. Therefore, hospital-acquired pneumonia is the most frequent clinical condition connected with these bacteria, particularly associated with patients receiving the mechanical ventilator assistance [5, 6]. In addition, *A. baumannii* can also cause blood stream infections, wound and urinary tract infections, and meningitis [7, 8]. Treatment of those infections, if caused by multidrug-resistant bacteria, involves the use of combinations of such agents as colistin, polymyxin B, and tigecycline. However, the safety of these approaches, even if successful, has not yet been well documented [9–11]. In this situation, the search for new, effective antimicrobials is demanded. Antimicrobial peptides are an interesting class of compounds that can be an alternative to conventional antibiotics. These endogenic molecules are widely distributed in nature, mainly as a part of innate immunity of organisms [12]. They target a broad spectrum of pathogens (bacteria, fungi, protozoa, and viruses) and are associated with triggering and coordinating multiple components of innate and immune adaptive systems [13–16]. Owing to these properties, antimicrobial peptides (AMPs) have attracted much attention of researchers from many scientific groups worldwide. Generally, AMPs are 11–50 amino acid residue long, amphipathic molecules with net positive charge [17, 18]. The main mechanism of their antimicrobial activity is based on a non-receptor-mediated microbial membrane disruption. However, they can also lead to microbial cell death through other mechanisms such as inhibition of protein/DNA or cell wall synthesis or induction of apoptosis/necrosis. Owing to the membrane-associated activity, they could also exhibit a low potency to trigger antimicrobial resistance [19]. The aim of this study was to evaluate the activity of eight AMPs, namely aurein 1.2, CAMEL, citropin 1.1., LL-37, omiganan, r-omiganan, pexiganan, and temporin A, against reference a strain of *A. baumannii* ATCC 19606. The microbiological studies involved the determination of MIC, MBC, and MBEC. Interestingly, the antibiofilm activity was evaluated both on a polystyrene surface and tracheal tube fragments. The results were correlated with those of conventional antibiotics: ciprofloxacin, erythromycin, gentamicin, piperacillin, rifampicin, and tigecycline. Cytotoxicity of tested peptides was determined by MTT assay.

## Materials and Methods

### Peptide Synthesis

Peptides used in this study (Table 1) were synthesized manually by solid-phase method using Fmoc chemistry on polystyrene resin modified by a Rink amide linker. Deprotection of the Fmoc group was carried out in a 20% (*v*/*v*) piperidine (Merck, Darmstadt, Germany) solution in DMF (Honeywell, Seelze, Germany) for 15 min. Acylation with protected amino acid was conducted in a DMF/DCM solution (Chempur, Piekary Slaskie, Poland) with coupling agents for 1.5 h using a threefold molar excess of DIC (Peptideweb, Zblewo, Poland) and OxymaPure (Iris Biotech GmbH, Marktredwitz, Germany). Every step was preceded by rinsing the resin and running the chloranil test. Peptides were cleaved from the resin using one of the mixtures: (A) TFA (Apollo Scientific, Denton, UK), phenol, TIS (Sigma-Aldrich, St. Louise, MO, USA), and water (92.5:2.5:2.5:2.5 *v*/*v*); (B) TFA, triisopropylsilane, and water (95:2.5:2.5 *v*/*v*). Mixture (A) was used with peptides containing a tryptophan residue, whereas mixture (B) with the remaining peptides. Crude peptides were precipitated with cold diethyl ether (Chempur, Piekary Slaskie, Poland) and lyophilized. Subsequently, the peptides were purified by RP-HPLC with LP-chrom software (Lipopharm.pl, Poland). Purifications were carried out on a Phenomenex Gemini-NX C18 column (21.20 × 100 mm, 5.0-μm particle size, 110-Å pore size). UV detection at 214 nm was used, and crude peptides were eluted with a linear 10–70% acetonitrile gradient in deionized water over 90 min at room temperature. The mobile phase flow rate was 10.0 mL/min. Both eluents contained 0.1% (*v*/*v*) of TFA. Fractions were analyzed on a Waters X-Bridge Shield RP-18 column (4.6 × 150 mm, 3.5-μm particle size, 130-Å pore size) with UV detection at 214 nm. Pure fractions (> 95%, by HPLC analysis) were collected and lyophilized. The identity of all compounds was confirmed by mass spectrometry (ESI-MS).

**Table 1:** Peptides used in this study

Name	Sequence
Aurein 1.2	GLFDIIKKIAESF-NH_2_
CAMEL	KWKLFKKIGAVLKVL-NH_2_
Citropin 1.1	GLFDVIKKVASVIGGL-NH_2_
LL-37	LLGDFFRKSKEKIGKEFKRIVQRIKDFLRNLVPRTES
Omiganan	ILRWPWWPWRRK-NH_2_
r-Omiganan	KRRWPWWPWRLI-NH_2_
Pexiganan	GIGKFLKKAKKFGKAFVKILKK-NH_2_
Temporin A	FLPLIGRVLSGIL-NH_2_

### Cultivation of Organisms

The *A. baumannii* ATCC 19606 strain was acquired from the American Type Culture Collection and prepared in line with the manufacturer’s instructions. The culture was stored in Roti®-Store Cryo-Vials (Carl Roth GmbH, Karlsruhe, Germany) at − 20 °C. Before the tests, the bead with cryo-protected bacteria was transferred into fresh MHB (Biocorp, Warsaw, Poland) and incubated for 24 h at 37 °C. Then, the culture was seeded on MHA plates and incubated as just mentioned. The agar cultures prepared in this way were used for further experiments. Cell densities for all assays were adjusted spectrophotometrically (Multiskan™ GO Microplate Spectrophotometer, Thermo Scientific) at 600 nm.

### Activity Against Planktonic Forms of Bacteria

The MICs and MBCs were determined with the reference to the Clinical and Laboratory Standards Institute guidelines [20]. MICs were determined by broth microdilution method. For this purpose, initial inoculums of bacteria (0.5 × 10^5^ CFU/mL) in MHB were exposed to a series of concentrations (0.5–256 μg/mL) of the compounds and incubated for 18 h at 37 °C. The experiments were conducted on 96-well microtiter polystyrene plates with the final volume of 100 μL. The MIC was taken as the lowest drug concentration at which a noticeable growth of microorganisms was inhibited. In parallel, the MIC wells, two above and one below (positive control), were cultured on a MHA plate. The lowest concentration of test compounds that did not show any visible growth of bacteria on the solid medium after 24 h of incubation at 37 °C were defined as MBC. All experiments were conducted in triplicate.

### Activity Against Biofilm

The antibiofilm activity was determined on 96-well flat-bottomed microtiter polystyrene plates and 0.5-cm fragments of tracheal tubes (RIM-75, ZARYS International Group, Zabrze, Poland), with resazurin (7-hydroxy-3H-phenoxazin-3-one 10-oxide) as a cell viability reagent. The first part included assay on 96-well plates. To do this, the plates were filled with 100 μL of the initial inoculums of bacteria (0.5 × 10^7^ CFU/mL) in the MHB and incubated for 24 h at 37 °C. Then, the wells were rinsed three times with PBS to remove non-adhered cells and the fresh medium with a series of concentrations (0.5–256 μg/mL) of the compounds was added. After 24 h of incubation, 20 μL of resazurin (4 mg/mL) was added to each well and the MBEC values were read. The assay on tracheal tube fragments was conducted using 24-well polystyrene plates. Briefly, the tracheal tube fragments were dipped in 1 mL of the bacterial inoculum (0.5 × 10^7^ CFU/mL) and incubated at 37 °C. After 24 h of incubation, the test fragments were rinsed three times with PBS and transferred into other wells containing the fresh medium with a series of concentrations of the test compounds. Subsequently, the plates were incubated again for 24 h at 37 °C. Then, 200 μL of resazurin was added and the MBEC was read. All experiments were conducted in triplicate.

### MTT Assay

To evaluate the cytotoxicity of the test peptides (IC_50_), the classic MTT assay on 96-well plates was performed for human keratinocytes (HaCaT) which were acquired from the ATCC. The assay utilizes colorimetric determination of the cell metabolic activity and the color intensity reflects the number of live cells that can be measured spectrophotometrically. The cell line was cultured in Dulbecco’s modified Eagle Medium (Invitrogen) supplemented with 10% fetal bovine serum (*v*/*v*), 100 units/mL of penicillin, 100 μg/mL of streptomycin, and 2 mM l-glutamine and was kept at 37 °C in a humidified 5% CO_2_ incubator. Briefly, a day after plating of 500 cells per well, a series of concentrations (0.5–500 μg/mL) of the test compounds were applied. DMSO was added to the control cells at a final concentration of 1.0% (*v*/*v*), which was related to the maximal concentration of the solvent compounds used in the experiment. After 24 h of incubation at 37 °C (humidified 5% CO_2_ incubator) with the peptides, a medium containing 1 mg/mL of MTT was added to the wells up to a final concentration of 0.5 mg/mL. Subsequently, the plates were incubated at 37 °C for 4 h. Then, the medium was aspirated, and the formazan product was solubilized with DMSO. The background absorbance at 630 nm was subtracted from that at 570 nm for each well (Epoch, BioTek Instruments, USA). Six replicates were conducted for each concentration. All experiments were repeated at least twice and the resulting IC50 values were calculated with a GraFit 7 software (v. 7.0, Erithacus, Berkley, CA, USA).

## Results

### Antimicrobial Assay

Eight different AMPs and six conventional antibiotics were tested against the reference strain of *A. baumannii* ATCC 19606. The results indicate that the peptides exhibit a high activity against planktonic forms of the bacteria (Table 2). The most effective was CAMEL and pexiganan with MIC and MBC values of 2 and 4 μg/mL, respectively. The next was LL-37 with an order of magnitude lower activity and citropin 1.1 with MIC and MBC values of 16 μg/mL. Interestingly, the identical activities exhibited aurein 1.2 and r-omiganan, the retro analog of omiganan, which turned out to be stronger than the parent compound (for which the MICs and MBCs were 32 and 64 μg/mL, respectively). Temporin A exhibited the highest minimum inhibitory and bactericidal concentrations of 128 μg/mL. In conclusion, the antimicrobial activity of the peptides decreased in the order: CAMEL, pexiganan > LL-37 > aurein 1.2, citropin 1.1, r-omiganan > omiganan > temporin A. Among the antibiotics tested, the most antimicrobially active was ciprofloxacin with MICs and MBCs of 0.5 and 1 μg/mL, respectively. With rifampicin and tetracycline, these quantities ranged between 2 and 4 μg/mL, while with erythromycin and gentamicin between 16 and 32 μg/mL. Piperacillin was the least effective antibiotic with MICs and MBCs of 64 and 128 μg/mL, respectively. In general, the antimicrobial activity of antibiotics decreased in the order: ciprofloxacin > tetracycline, rifampicin > gentamicin, erythromycin > piperacillin.

**Table 2:** Antimicrobial activity of the test compounds [μg/mL] against *A. baumannii* ATCC 19606

Compound	MIC	MBC	MBEC	MBEC^tt^
Ciprofloxacin	0.5	1	2	1
Erythromycin	32	32	32	16
Gentamicin	16	32	32	32
Piperacillin	64	128	> 512	> 512
Rifampicin	4	4	8	4
Tetracycline	2	4	16	4
Aurein 1.2	16	16	64	32
CAMEL	2	4	128	64
Citropin 1.1	16	16	256	32
LL-37	4	8	512	128
Omiganan	32	64	256	128
r-Omiganan	16	16	256	128
Pexiganan	2	4	256	32
Temporin A	128	128	512	256

### Antibiofilm Activity

Antibiofilm studies and the results of MBEC indicate that eradication of biofilm from tracheal tubes occurred as a rule at lower concentrations than that of the polystyrene surface of microtiter plates (Table 2). However, the tested AMPs appear to exhibit a lower antibiofilm activity than did conventional antibiotics. The most active compound was ciprofloxacin with MBEC values for polystyrene and tracheal tubes of 2 and 1 μg/mL, respectively. Interestingly, erythromycin eradication concentrations were identical to those of MIC and MBC and by an order of magnitude lower for tracheal tubes. The antibiofilm activity of gentamicin and rifampicin appears to be almost equal for planktonic cultures while with tetracycline, this activity was found to be threefold lower. Among all the test compounds, only piperacillin failed to eradicate the biofilm at any concentration. The most active peptide was aurein 1.2 with up to twice as high MBEC than MIC. On the other hand, the largest difference between MICs and MBECs was found for CAMEL (the sixfold lower activity), pexiganan, and LL-37 (the sevenfold lower activity). What is more, identical MBECs were found for omiganan and its retro analog. Temporin A was up to twice less active against biofilm, but the concentrations were relatively high (512 and 256 μg/mL for biofilm on the polystyrene and tracheal tubes, respectively). In general, the MBEC^tt^ were either lower (up to threefold in magnitude) or equal to MBEC for all of the test compounds (antibiotics and peptides).

### Cytotoxicity

Evaluation of cytotoxicity of the majority of peptides used in this study was conducted by us previously and the present analyses were run for omiganan and r-omiganan only [21]. Both the previous and actual results were used for calculation of SI (Table 3). Based on the cumulative set of the IC_50_ values, it can be stated that the majority of peptides exhibited relatively high toxicity against HaCaT cell line. The IC_50_ values ranged between 1.05 and 79.39 μg/mL and for half of them were lower than the MIC (the exception was found for CAMEL, LL-37, omiganan, and r-omiganan). Interestingly, the IC_50_ for LL-37 was nearly three times lower than the MIC value (SI = 2.93).

**Table 3:** IC_50_ values and SI of the peptides

Compound	IC_50_	MIC	SI (IC_50/_/MIC)
CAMEL	3.32^*^	2	1.66
Citropin 1.1	3.02^*^	16	0.18
LL-37	11.75^*^	4	2.93
Omiganan	79.39	32	2.48
r-Omiganan	29.51	16	1.84
Pexiganan	1.05^*^	2	0.52
Temporin A	7.91^*^	128	0.06

^*^These analyses were performed by our group in the previous study [21]

## Discussion

Recent scientific reports highlight the problem of an increasing resistance to antibiotics and global spread of multidrug-resistant bacteria. For this group, a special term of ESKAPE pathogens has been proposed, which strictly refers to specific species namely, *Enterococcus faecium*, *Staphylococcus aureus*, *Klebsiella pneumoniae*, *Acinetobacter baumannii*, *Pseudomonas aeruginosa*, and *Enterobacter* [22]. As these bacteria are frequently isolated from patients with severe, difficult-to-treat infections, new therapeutic options are needed. Moreover, the research on new antimicrobial compounds should be focused on this particular group of pathogens with orientation on reference as well as clinical isolates and the activity against biofilm. It is commonly known that survival potential of bacteria is enhanced by biofilm formation, but this feature is not always taken into account in the clinic. In fact, the susceptibility profile of bacteria is routinely determined for planktonic cultures. However, biofilm can be described as a cellular conglomerate attached to a surface (both biotic and abiotic) enclosed in a matrix built up with extracellular polymeric substance (EPS) [23, 24]. As compared to the freely suspended planktonic cultures, this structure is 10 up to 1000 times more resistant to antimicrobials. Particularly noteworthy is the fact that the biofilm is responsible for over 80% of microbial infections in the body. Moreover, biofilm-producing bacteria have a capability to colonize indwelling devices, thus leading to infections [24–26]. *A. baumannii* is one of the strains that causing medical device colonization becomes one of the major sources of systemic dissemination [27]. AMPs as compounds with great antibiofilm potential can find application in the treatment of *A. baumannii* infections [28–32]. In this study, eight AMPs, namely, aurein 1.2, CAMEL, citropin 1.1., LL-37, pexiganan, omiganan, r-omiganan, and temporin A, were tested using reference strain of *A. baumannii* ATCC 19606. Furthermore, the antibiofilm activity was also determined for tracheal tube fragments. This approach is quite unique since medical devices are used more likely for the analysis of bacteria or their isolation [33–35]. As a matter of fact, the growth of *A. baumannii* on tracheal tubes is one of the major sources of colonization in lungs and bacterial pneumonia [36, 37]. Among the tested peptides, CAMEL and pexiganan displayed the highest activity with MIC values of 2 μg/mL. These results are consistent with other studies conducted on clinical strains of *A. baumannii*. For example, Giacometti et al. conducted a research on in vitro activity of cecropin A, melittin, and CAMEL [CA(1–7)M(2–9)NH_2_], alone and in combination with antibiotics, against 32 nosocomial isolates of *A. baumannii* [38]. The MICs and MBCs ranged between 0.25–8 and 0.5–16 μg/mL, respectively. In spite of the fact that the peptide was found to be very active with the potential in adjuvant therapy with other antibiotics, the authors suggested that further studies on safety determination had to be conducted. Pexiganan is another peptide that was previously tested against clinical isolates. For instance, in the study conducted by Ge et al., susceptibility profiles of a number of strains were determined [39]. The distribution of the MICs ranged between 1 and 8 μg/mL and the value of 2 μg/mL was found for the majority of strains (49 isolates). Furthermore, Flamm et al. conducted a research on the activity of pexiganan against pathogens isolated from diabetic foot infection (DFI). [39]. The study involved not only bacteriological assays, but also characterization of resistance genes. The MICs for two DFI-associated *A. baumannii* strains were equal to 8 μg/mL. Moreover, pexiganan and colistin were also applied in experimental mouse models of *A. baumannii* infection [40]. In that study, the compounds were used for susceptibility determination and survival assessment of mice. It should be noticed that pexiganan was found to be effective in combinatory approach (low bacterial count) and also increased NK cytotoxic activity over the levels of infected and colistin-treated animals. LL-37 is the third most active peptide assessed in that study and, at the same time, the most extensively studied one against *A. baumannii* [41–44]. In the research conducted by Feng et al., antibacterial and antibiofilm activities of LL-37 and its fragments (KS-30, KR-20, KR-12) against clinical strains were determined [41]. Furthermore, not only MICs but also the kinetics, impact on biofilm formation, development, and dispersal were assessed. Although the MICs for the clinical strains applied in their study were higher than those obtained in this research (16 and 32 μg/mL versus 4 μg/mL), the impact on biofilm formation occurred in similar concentrations. Our study revealed that LL-37 eradicates biofilm at a very high concentration (MBEC = 512 μg/mL). This suggests that application of this peptide is much more recommendable for prevention of biofilm-associated infections than for their treatment. Aurein 1.2 and citropin 1.1 are the peptides of amphibian origin that in our study exhibited identical activities against planktonic forms of *A. baumannii* (16 μg/mL) and the biofilm on tracheal tubes (32 μg/mL). It should be emphasized that the MBEC value obtained on polystyrene plates was four times lower for aurein 1.2 (256 versus 64 μg/mL). Despite the fact those peptides were extensively studied for their antimicrobial and physicochemical properties, their impact on *A. baumannii* has not been [45–50]. Only aurein 1.2, in a study conducted by de Freitas et al. on the enhancement of antimicrobial photodynamic therapy, has been found to reduce significantly the viability of *S. aureus*, *Escherichia coli*, and *A. baumannii* as well [51]. Omiganan and its retro analog (r-omiganan) were also tested in our study; however, only the first one has been studied against *A. baumannii* so far. In a study conducted by Sader et al., the antimicrobial activity of omiganan pentachloride (formerly MBI 226) was determined against more than one and a half thousand clinical isolates of bacteria and yeasts [52]. Moreover, not only the susceptibility profiles to antimicrobial peptide and conventional antibiotics was assessed, but also killing kinetics and precise quantification of resistant strains were estimated. The MIC obtained with the strain *A. baumannii* 101-2823A for omiganan was 8 μg/mL, which is twice lower than that determined in our study. In addition, r-omiganan was found to be a stronger antimicrobial than the parent compound. The results are consistent with our previous work where the retro analog of omiganan exhibited improved activity against Gram-negative strains as compared to that of the parent peptide. At the same time, omiganan is less cytotoxic than its retro counterpart owing to the lower rate of hemolysis. [53]. Temporin A was the weakest peptide tested in our study and the most cytotoxic one. The activity against planktonic forms was 128 μg/mL, which was 16 times higher than the IC_50_. Even more, the MBEC values for tracheal tube fragments and polystyrene were twice and four times higher, respectively. As a matter of fact, temporin A was previously tested against *A. baumannii*. In a study by Mangoni et al., temporins A, B, and G were tested against multidrug-resistant clinical isolates of multiple bacterial strains involved in nosocomial infections. The results reported by this group indicated that the *A. baumannii* strains were sensitive to temporins over a concentration range comparable or lower than that required to kill Gram-positive strains such as *S. aureus*. However, these results are inconsistent with our findings, due to the different protocols of antimicrobial assay as well as the use of human serum in antimicrobial determination. Selectivity indexes (Table 3) calculated for all peptides suggest that citropin 1.1, pexiganan, and temporin A are distinctly cytotoxic (HaCaT) and MIC is relatively high (SI between 0.06 and 0.52). The highest SIs were determined for LL-37 and omiganan (2.93 and 2.48, respectively). Moreover, retro omiganan and CAMEL have slightly lower SIs (1.84 and 1.66, respectively). Nevertheless, different methods, particularly the materials, used for determination of antimicrobial activity can also give inconsistent results. This should be highlighted that positively charged molecules like AMPs may bind to the polystyrene surface. In our study, the polystyrene plates were used for determination of MIC and MBEC as well. For instance, Giacometti et al. have compared the activity of eight AMPs using two protocols, the first described by CLSI (formerly NCCLS) and the second by R.E.W. Hancock, which utilizes the use of polypropylene instead [54]. The results highlighted significant differences between those methods and indicated the higher activity of AMPs when the polypropylene plate was used. However, opposite observations were found in the study of Sánchez-Gómez et al. [55]*.* Therefore, it is reasoned to evaluate the activity of AMPs against *A. baumannii* on polystyrene and polypropylene plates in further studies.

## Conclusions

Despite the fact that the peptides used in this study, except temporin A, were found to be effective against planktonic forms of the reference strain *A. baumannii* ATCC 19606, their activity against biofilm formed on polystyrene and tracheal tube fragments was distinctly lower, especially as compared to the activities of conventional antibiotics. Nevertheless, it should be emphasized that the main characteristic of *Acinetobacter* spp. is their capability to acquire resistance. In fact, there is a risk that the compounds being effective against the reference strains may be inapplicable clinically. For this reason, the therapy of many infections caused by these bacteria is so challenging. Our study revealed how difficult is to eradicate the *A. baumannii* biofilm and indicated the difference between the characters of the surface where it has been formed. The peptides tested in our study turned out to be weaker antimicrobials than antibiotics. However, it should be noted that peptides as positively charged molecules may bind to the surface of polystyrene and therefore, their antimicrobial activity may be underestimated. This notwithstanding, the compounds constitute a suitable basis for the design of new compounds with improved activity. Moreover, they proved to be more effective in combinatory approach. Many studies have shown their capability of binding to indwelling devices, thus making them resistant to biofilm formation. That is why the research on this particular group should be continued, with the focus on their biological and physicochemical characterization.

## Abbreviations

AMPs: Antimicrobial peptides
ATCC: American Type Culture Collection
CFU: Colony-forming units
DCM: Dichloromethane
DIC: *N*,*N*′-diisopropylcarbodiimide
DMF: *N*,*N-*dimethylformamide
DMSO: Dimethyl sulfoxide
ESI-MS: Electrospray ionization mass spectrometry
Fmoc: N-9-fluorenylmethoxycarbonyl
HPLC: High-performance liquid chromatography
MBC: Minimum bactericidal concentration
MBEC: Minimum biofilm eradication concentration
MBEC^tt^: Minimum biofilm eradication concentration on tracheal tube fragments
MHA: Mueller-Hinton agar
MHB: Mueller-Hinton broth
MIC: Minimum inhibitory concentration
MTT: 3-(4,5-Dimethyl-2-thiazolyl)-2,5-diphenyl-2-H-tetrazoluim bromide
PBS: Phosphate-buffered saline
RP-HPLC: Reversed-phase liquid chromatography
SI: Selectivity index
TFA: Trifluoroacetic acid
TIS: Triisopropylosilane
